# Detergent-free Ultrafast Reconstitution of Membrane Proteins into Lipid Bilayers Using Fusogenic Complementary-charged Proteoliposomes.

**DOI:** 10.3791/56909

**Published:** 2018-04-05

**Authors:** Mikhail A. Galkin, Aidan N. Russell, Steven B. Vik, Richard M. Berry, Robert R. Ishmukhametov

**Affiliations:** ^1^First Pavlov State Medical University; ^2^Clarendon Laboratory, Department of Physics, Oxford University; ^3^Department of Biological Sciences, Southern Methodist University

**Keywords:** Retraction, Issue 134, Membrane proteins, reconstitution, proteoliposomes, LUV, GUV, membrane fusion, electron transport chain, ATP synthase, ATP production, synthetic biology

## Abstract

Detergents are indispensable for delivery of membrane proteins into 30-100 nm small unilamellar vesicles, while more complex, larger model lipid bilayers are less compatible with detergents.

Here we describe a strategy for bypassing this fundamental limitation using fusogenic oppositely charged liposomes bearing a membrane protein of interest. Fusion between such vesicles occurs within 5 min in a low ionic strength buffer. Positively charged fusogenic liposomes can be used as simple shuttle vectors for detergent-free delivery of membrane proteins into biomimetic target lipid bilayers, which are negatively charged. We also show how to reconstitute membrane proteins into fusogenic proteoliposomes with a fast 30-min protocol.

Combining these two approaches, we demonstrate a fast assembly of an electron transport chain consisting of two membrane proteins from *E. coli*, a primary proton pump bo_3_-oxidase and F_1_F_o_ ATP synthase, in membranes of vesicles of various sizes, ranging from 0.1 to >10 microns, as well as ATP production by this chain.

**Figure:**
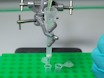


## Introduction

Functionalization of artificial lipid bilayers with membrane proteins is a key step in assembly of membrane model systems. The simplest model, proteoliposomes (PL), consists of small (30-200 nm diameter) unilamellar vesicles (SUV, also called liposomes), with proteins integrated into their membranes. PL are traditionally formed in two steps^1^. First, preformed SUV are mixed with a membrane protein of interest and a detergent at a concentration above its critical micelle concentration (CMC). Second, the detergent is removed with various dialysis, "bio-beads" or gel filtration techniques, leaving the protein in the membrane. The latter approach is much faster (~30 min^1^) and is therefore preferable for reconstitution of fragile and sensitive membrane proteins, while the first two approaches are limited by detergent removal speed, which takes many hours and may cause a substantial loss of activity and loss of structural integrity of the proteins. Functionalization of larger vesicles (large unilamellar vesicles, LUV, up to 1 µm diameter) by this approach is more challenging, as vesicle size gets reduced after detergent removal, and it is not possible for giant unilamellar vesicles (GUV, >1 µm), as they are destabilized by detergents (but see Johnson *et al.*^2^ for slow 2D-crystallization of membrane proteins in large bilayers). Alternative approaches for GUV membrane functionalization^3^,^4^,^5^ exist but are laborious, time consuming, and still require some detergent at concentrations below CMC. More complex or fragile lipid models (for example, Droplet Hydrogel Bilayers^6^ and 3D printable Droplet Interface Bilayer-based artificial tissues^7^) cannot tolerate detergents. Quickly emerging synthetic biology applications^8^,^9^,^10^ critically depend on functionalization of such complex membrane structures. Therefore, an easy and robust method allowing fast and gentle delivery of membrane proteins into the target fragile bilayers is highly sought in the field.

An alternative, detergent-free protein delivery method is vesicle fusion, where interacting vesicles' membranes unite into the intact postfusion bilayer, while their intravesicular aqueous contents get mixed, without being released into the external environment. Vesicle fusion is enabled and driven either by conformational rearrangements within complementary fusogenic agents (some proteins^11^,^12^ and peptides^13^ or specially modified DNA^14^) located in the contacting bilayers, or Coulombic interactions between lipid bilayers formed of complementarily charged cationic and anionic lipids^15^,^16^, or cationic bilayers and negatively charged proteins^17^.

The former approach requires the presence of fusogenic agents in the interacting membranes prior to fusion, is relatively slow (~30 min to reach half-maximum of fusion^12^,^18^), but can be applied to both natural and artificial membranes.

An advantage of the approach using fusogenic lipids (Figure 1) is that it enables much faster membrane fusion (~1 min to reach half-maximum, and 5-10 min to finish the reaction). Additionally, the extent of fusion can be controlled by i) an easy to formulate relative content of charged lipids in fusogenic bilayers, and ii) ionic and, in general, osmotic strength of the reaction medium (salts at above 50 mM and, for example, sucrose^15^ are shown to stop fusion), or a combination of both. To initiate fusion, oppositely charged fusogenic vesicles are mixed in a low (typically 10-20 mM salt) ionic strength medium for 5-10 min. A relative disadvantage of the method is that cationic lipids may exert a negative effect on functionality of membrane proteins in cationic proteoliposomes prior to fusion, especially in low ionic strength, but this effect is reversible and mitigated by a natural lipid composition of the post-fusion membrane and its return to the normal ionic strength medium.

## Protocol

### 1. Preparation of fusogenic SUV and LUV

1. **Preparation of fusogenic lipid mixture**
  1. Prepare stock solutions of neutral, cationic, anionic, and fluorescent lipids in chloroform at 25-50 mg/mL (for example, neutral DOPC (1,2-dioleoyl-sn-glycero-3-phosphocholine), cationic ethyl-PC (1,2-dimyristoleoyl-sn-glycero-3-ethylphosphocholine), anionic POPA (1-palmitoyl-2-oleoyl-sn-glycero-3-phosphate), and fluorescent cholesteryl-Bodipy-FL12, respectively) by weighing appropriate amounts of dry lipids and dissolving them in 100% chloroform (Caution! Do this under a fume hood), or diluting commercially available chloroform stocks of lipids with chloroform. NOTE: Both natural lipid extracts and pure synthetic lipids can be used and demonstrate similar results.
  2. Mix 2.5 mg a cationic and 2.5 mg of a neutral lipid in chloroform stock (1.1.1) in a glass vial. NOTE: This step yields 5 mg of cationic lipid mixture in chloroform, containing 50% weight fraction of cationic lipids. When needed, add 0.5% weight fraction of a fluorescent lipid to the mixture.
  3. Mix 1 mg of anionic and 4 mg of neutral lipids in chloroform stocks (1.1.1) in a glass vial. This gives 5 mg of anionic lipid mixture containing 20% weight fraction of anionic lipids.
  4. Evaporate chloroform under a stream of nitrogen to form a thin layer of dry lipid.
  5. Remove the residual chloroform under vacuum for 10 min. NOTE: Traditionally this step takes much longer (1-12 h), but we found that a longer treatment provides no obvious improvement in coupling properties of SUV.
  6. Hydrate the dry lipid film by adding 0.5 mL **buffer A** (100 mM KCl, 1 mM MgCl_2_, 50 mM MOPS, pH 7.4) to each vial and leaving them to stand at room temperature for 30 min, and then vortex until the lipid film is completely detached from the glass surface and becomes a homogenous lipid suspension. This should take about 20 - 40 s.
2. **Formation of SUV and LUV by extrusion**
  1. Assemble an extrusion system (see the **Table of Materials**) with two 1 mL syringes using a polycarbonate filter with one of the following pore sizes: 100 or 200 nm to form SUV, and 400 or 800 nm to form LUV.
  2. Transfer the lipid suspension into a syringe.
  3. Extrude the suspension by passing it through the filter 21 times, as shown elsewhere^18^,^19^. NOTE: An odd number of passages is needed to minimize transfer into the vesicle solution of large lipid clumps stuck to the filter side facing the original lipid suspension.
  4. Transfer vesicle solution into 1.5 mL microcentrifuge tubes.

### 2. Formation of Fusogenic GUV by Inverted Emulsion Method

NOTE: This procedure is illustrated in Figure 2.

1. **Preparation of lipid-in-oil solution**
  1. Mix a chloroform stock (see step 1.1.1) of a neutral lipid (2 mg) and an anionic lipid (0.5 mg) with 1 mL of hexadecane in 1.5-mL microcentrifuge tube.
  2. Evaporate chloroform from the mixture under constant mixing and heating at 80 °C for 30 min keeping the tube open. Close the tube and let cool to room temperature.
2. **Formation of a lipid monolayer at the lipid-in-oil/aqueous buffer interface**
  1. Place 200 µL of the lipid-in-oil solution on top of 0.5 mL **buffer B** (20 mM KCl, 0.1 mM MgCl_2_, 10 mM MOPS pH 7.4) in a 1.5 mL centrifuge tube. Note the convex shape of the phase separation border due to surface tension mismatch between the oil and the aqueous buffer (Figure 2A).
  2. Wait until the phase separation border flattens, which is indicative of lipid monolayer formation on it. This typically takes 30 - 60 min at room temperature.
3. **Preparation of a water-in-oil emulsion**
  1. Prepare a solution of a water-soluble non-ionic polysaccharide in buffer B, with a density higher than density of water (*e.g.* 15% Ficoll-400 (w/v), with density 1.05 g/mL) NOTE: Optionally, one can add fluorescent water-soluble dyes to the buffer for better visualization of GUV, and any other desired aqueous contents of the GUVs.
  2. Transfer 0.5 µL of the above mixture to a separate 1.5-mL centrifuge tube with 100 µL of the lipid-in-oil solution from 2.1.2.
  3. Sonicate (44 kHz at 14 W) the mixture for 30 s in an ultrasonic water bath. Then, vigorously vortex for 45 min to form a water-in-oil emulsion, where each droplet will be coated by a lipid monolayer (Figure 2B).
4. **Conversion of the water-in-oil emulsion into GUV**
  1. Place the resultant emulsion on top of the lipid-in-oil/aqueous interface, and immediately centrifuge the tube in a table-top centrifuge at 10,000 x g for 2 min. The resultant pellet of GUV should be clearly visible (Figure 2C).
  2. Cool the tube to 4 °C in a refrigerator to solidify the lipid-in-oil mixture (Figure 2D, hexadecane solidifies below 18 °C), carefully remove frozen oil, and then withdraw the aqueous phase. NOTE: To facilitate oil removal freeze a wire bent in the shape of an anchor was used.
  3. Resuspend the GUV pellet in 50 µL of fresh buffer B, transfer to a fresh tube, and inspect under a fluorescence microscope using a 100X oil immersion objective (Figure 2E).

### 3. Monitoring Vesicle Fusion with Cobalt-Calcein Method

1. **Preparation of a gel-filtration gravity column.**
  1. Soak ~10 g of a gel-filtration resin (*e.g.* superfine sephadex G-50) in 100 mL of deionized water, and let swell overnight.
  2. Pack 3 mL of the resin into a disposable plastic gravity flow column, wash it with ultrapure water, and equilibrate with **buffer C** (100 mM KCl, 10 mM MOPS, pH 7.4) at room temperature.
2. **Preparation of SUV^+^ or SUV^0^ loaded with cobalt-calcein.**
  1. Prepare a solution containing 1 mM calcein, 1 mM CoCl_2_, 98 mM NaCl, 10 mM MOPS, then bring pH to 7.4. NOTE: The essence of the method is that free fluorescent calcein forms a non-fluorescent complex with Co^2+^. It becomes fluorescent again upon addition of EDTA, which having higher affinity for Co^2+^, displaces calcein from the cobalt-calcein complex (Figure 3A).
  2. Add 500 µL of this solution to a dry film of cationic or neutral lipids prepared as described in 1.1.
  3. Prepare 100 nm SUV by extrusion as described in 1.2.
  4. Pellet extruded SUV at 1,000,000 x g for 20 min in a table-top ultracentrifuge and resuspend in 1 mL of **buffer C**. Repeat pelleting and resuspension three times; use 0.6 mL of buffer C for the last resuspension. NOTE: These steps remove most of the external cobalt-calcein.
  5. Remove the remaining external cobalt-calcein by passing SUV through a disposable gravity-flow column loaded with the gel-filtration resin equilibrated with buffer C as described for step 3.1.2. NOTE: We typically discard the first milliliter volume of the flow-through and collect the second milliliter, which contains external cobalt-calcein free SUV.
3. **Preparation of SUV^-^ loaded with EDTA**
  1. Prepare solution of 10 mM EDTA, 80 mM NaCl, 10 mM MOPS, pH 7.4.
  2. Add 500 µL of this solution to a dry film of anionic lipids prepared as described in 1.1.
  3. Prepare SUV^-^ by extrusion as described in 1.2.
  4. Pellet and resuspend SUV^-^ as described in 3.2.4.
  5. Remove the remaining EDTA by passing SUV^-^ through the gel-filtration column as described in 3.2.5.
4. **Preparing SUV for fusion**
  1. Dilute SUV^0^, SUV^+^, and SUV^-^ with **buffer D** (1 mM MOPS, pH 7.4) supplemented with 0.2 mM CoCl_2_ and the desired concentration of KCl. Use 5 µL of each type of SUV per 1 mL of buffer D.
  2. Incubate the mixture for at least 1 h. NOTE: This step will minimize the background fluorescence level by blocking calcein that is surface-bound to SUV^+^.
5. **Vesicle fusion**
  1. Start fusion reaction by mixing 1 mL of SUV^+^ or SUV^0^ with 1 mL of SUV^−^ (diluted in buffer D as described above) in a 2-mL fluorimeter cuvette, and monitor increasing fluorescence of calcein using 480 nm excitation and 510 nm emission (Figure 3B).
  2. Wait until the reaction comes to completion.
  3. Add a mixture of detergent Triton X-100 and EDTA (0.05% final concentration and 7 mM, respectively) to release calcein out of vesicles and obtain the maximum fluorescence signal.
  4. Determine the extent of fusion defined as the percentage of the maximum fluorescence signal following the detergent addition as shown in Figure 3C.

### 4. Fast Reconstitution of Membrane Proteins into Fusogenic Proteoliposomes

NOTE: This procedure is illustrated in Figure 4A.

1. Prepare a gravity flow column with the gel-filtration resin as described in 3.1, and equilibrate it with buffer A at room temperature. NOTE: All further manipulations must be done **strictly at ≤ 4 °C**, unless otherwise indicated, to minimize loss of protein activity.
2. Adjust concentration of the purified membrane protein to 0.7 mg/mL by diluting it with the same extraction buffer used for protein isolation.
3. Mix 140 µL of the protein with 300 µL of preformed SUV, 160 µL buffer A, and 60 µL of 10% cholate in Buffer A; this gives 1:30 protein: lipid weight ratio in a final 660 µL volume.
4. Gently shake the mixture on a rocking platform for 15 min. NOTE: The following steps can be done at room temperature.
5. Pass the mixture through gel-filtration resin, and collect turbid fractions containing proteoliposomes.
6. Pellet proteoliposomes with a table-top ultracentrifuge at 400,000 x g for 15 min.
7. Discard the supernatant containing non-reconstituted protein, and resuspend the pellet in 1 mL of fresh buffer A.
8. Determine the protein content in PL and the supernatant using the Amido-black method^20^.
9. Determine the yield of reconstitution, which typically is around 50-70%. NOTE: Steps 4.6-4.8 may optionally be replaced by passing PL solution through 1 mL of Ni-NTA resin packed into a disposable gravity column and washed with Buffer A as described in 3.1.2. Such passing will separate non-reconstituted protein, which will preferentially be bound to the resin, from PL, most of which will be in the flow-through.

### 5. Functional Tests of Protein Activity in Proteoliposomes

NOTE: Both proteins used in this study are powerful proton pumps, which pump H^+^ into proteoliposomes upon addition of their substrates (Coenzyme Q_1_ for bo_3_-oxidase, and ATP for F_1_F_o_, respectively), thus building a proton gradient across the membrane (ΔpH). In case of successful co-reconstitution by fusion, such acidification can be observed as a decrease in fluorescence of a pH-sensitive probe ACMA (9-Amino-6-Chloro-2-Methoxyacridine)^12^,^15^, which is used routinely for such tasks (Figure 4B**-C**). Additionally substrate-specific activity of the proteins (Coenzyme Q_1_ oxidation by bo_3_-oxidase^22^ and ATP hydrolysis by F_1_F_o_^23^,^24^) can be monitored spectrophotometrically using various methods. Here, we demonstrate ATP hydrolysis activity of F_1_F_o_ PL using an ATP regenerating assay (Figure 4D), where two enzymes (pyruvate kinase (PK) and lactate dehydrogenase (LDH) maintain a constant concentration of ATP as follows. PK recycles ATP by converting ADP produced by F_1_F_o_ back into ATP at the expense of its substrate phosphoenolpyruvate (PEP). Pyruvate, which is a product of this reaction, is converted into lactate by LDH at the expense of NADH, the oxidation of which can be monitored as decreasing optical density at 340 nm.

1. **Preparation of chemicals**
  1. Prepare 1 mM ACMA stock in ethanol.
  2. Prepare a 1 mM stock of nigericin (or any other uncoupler) in ethanol.
  3. Prepare 25 mM Coenzyme Q_1_ stock in ethanol, and 1 M DTT stock in buffer A.
  4. Prepare a stock of 100 mM ATP in Buffer A, and adjust its pH to 7.4.
  5. Prepare stocks of ATP regenerating system components in Buffer A: 100 mM PEP, 1 mM NADH, and solutions of PK and LDH at concentration of ~500-1,000 units/mL.
2. **Coenzyme Q_1_ oxidation-driven proton pumping by bo_3_-oxidase PL**
  1. Add 20 µL of PL to a fluorimeter cuvette with 2 mL buffer A in presence of 0.5 µM ACMA, and wait until stable signal using 430 nm excitation and 515 nm emission.
  2. Add 40 µM Coenzyme Q_1_ to the cuvette.
  3. Initiate proton pumping by adding 2 mM DTT to PL (Figure 4B). NOTE: DTT reduces oxidized Coenzyme Q_1_ and makes it available to bo_3_-oxidase.
  4. Dissipate formed ΔpH by adding 2 µM uncoupler (for example nigericin). ACMA fluorescence signal should quickly return to nearly the original level. NOTE: There should be no ACMA quenching upon addition of the substrate, if the uncoupler is present in the reaction mixture initially.
3. **ATP hydrolysis-driven proton pumping by F_1_F_o_PL**
  1. Add 40 µL of PL to a fluorimeter cuvette as described in 5.2.
  2. Initiate proton pumping by adding 0.2 mM ATP to PL (Figure 4C).
  3. Dissipate ΔpH as described in 5.2.4.
4. **ATP hydrolysis by F_1_F_o_PL, and its stimulation by the uncoupler**
  1. Add 40 µL of PL to a spectrophotometer cuvette with 2 mL buffer A, containing 1 mM ATP, 0.2 mM NADH, 2 mM PEP, and 15 µL each of PK and LDH.
  2. Follow the reaction by measuring absorbance decrease of NADH at 340 nm. 3-4 min later, add 2 µM uncoupler to the cuvette to release the backpressure of ΔpH on ATP hydrolysis. The reaction speed should increase immediately. NOTE: PL passed through Ni-NTA resin as described in 4.10 will demonstrate the strongest stimulation by the uncoupler (Figure 4D, red trace), while the eluate will be barely stimulated (blue trace).

### 6. Testing Influence of Lipid Environment and Ionic Strength on Functionality of Membrane Proteins in Fusogenic Proteoliposomes

1. Assess proton pumping by 40 µL of PL^+^, PL^-^ and PL^0^ with ACMA quenching assay in 2 mL buffer D supplemented with 1 mM MgCl_2_ and 20 (Figure 5, Panel A) or 100 mM KCl (Panel B) as described in 5.2 or 5.3 (Figure 5, red, black and blue traces).
2. Fuse 40 µL of PL^+^ with same volume of LUV^-^ in 2 mL of buffer D supplemented with 20 mM KCl, and test for ACMA quenching (green trace).
3. Run control experiments as in Step 2 by mixing, for example, PL and SUV of the same charge (grey trace). NOTE: Proton pumping should be improved by postfusion PL.

### 7. Delivery of Membrane Proteins into LUV and GUV by Fusogenic Proteoliposomes

1. Fuse 50 µL of PL^+^ with 50 µL of 800 nm LUV^-^ in 1 mL of buffer D supplemented with 1 mM MgCl_2_, and 20 mM KCl for 5 min.
2. Pellet the vesicles at 6,000 x g for 5 min, and discard supernatant containing unreacted PL^+^. Resuspend the pellet in 1 mL of buffer D supplemented with 1 mM MgCl_2_ and 100 mM KCl, and repeat pelleting and resuspending twice more.
3. Run ACMA quenching assay as described in 5.2 or 5.4.
4. Run control experiments, as in Steps 1-3, using combinations of complementary charged vesicles in high salt (200 mM KCl), non-fusogenic vesicles, and just empty LUV^-^ without PL^+^. NOTE: Proton pumping by postfusion LUV should be detected only when complementarily charged vesicles were used as shown in Figure 6.

### 8. Assembly of Electron Transport Chain from Individual Components in Membranes of LUV and GUV, and ATP Production by this Chain.

NOTE: A schematic of this procedure is illustrated in Figure 7A. ATP produced in this experiment will be registered by the luciferin-luciferase system, where conversion of synthesized ATP into pyrophosphate and AMP by luciferase is followed by light emission registered with a luminometer. We recommend using a single-tube luminometer, instead of the less sensitive microplate luminometers.

1. Mix 3 µL of bo_3_-oxidase PL^+^ and 5 µL of F_1_F_o_ PL^+^ formed as described in Section 4.
2. Fuse them with 3 µL of LUV or GUV^-^ (formed as described in Section 2) in 800 µL of buffer D supplemented with 20 mM KCl and 1 mM MgCl_2_ for 5 min.
3. Using high concentration stocks, add KCl and MOPS to 100 mM and 50 mM final concentration to the postfusion membranes. NOTE: In case of post-fusion LUV, this step will prevent their swelling due to osmotic offset between the external (buffer A) and the intravesicular (buffer D) salts concentration.
  1. Optionally, pellet the postfusion membranes as described in 7.2 to separate unreacted SUV^+^, and resuspend the pellet in 800 µL of buffer A.
4. Prepare 200 µL of a luciferin-luciferase-ADP cocktail containing 400 µM ADP, 50 µM luciferin, and 2.5 µg luciferase in buffer A
5. Add the cocktail to the postfusion mixture from Step8.3.
6. Add 40 µM of oxidized Coenzyme Q_1_, and trigger energization of the postfusion membranes by bo_3_-oxidase by adding 2 mM DTT. Wait for 1 min.
7. Initiate ATP synthesis by adding 5 mM potassium phosphate (KP_i_, pH 7.4) to energized vesicles, and detect ATP production with a luminometer (Figure 7B). Run the reaction for ~3 min. NOTE: ATP synthesis reaction proceeds for 5-7 min until depletion of oxygen consumed by bo_3_-oxidase and luciferase.
8. Add ATP reference standard (0.5 nmol ATP) to the reaction mixture twice. Measure ATP produced.
9. Obtain the actual amount of ATP produced in the reaction by dividing the signal from ATP synthesis reaction by the ATP reference standard signal. Adjust this value to the total amount of F_1_F_o_ used in the reaction, and finally express the rate of ATP synthesis in µmol ATP/(mg F_1_F_o_*min).

## Representative Results

The use of fusogenic complementary-charged proteoliposomes for fast detergent-free delivery of membrane proteins into target bilayer membranes includes three steps (Figure 1): **A**, formation of fusogenic SUV from lipid mixtures with high content of charged lipids; these SUV may optionally carry an intravesicular load; **B**, conversion of fusogenic SUV into PL using our fast membrane protein reconstitution; **C**, fusion of fusogenic PL with target bilayers in a low-salt medium, followed by addition of high salt to stop the fusion reaction. In case of large vesicles (**D**), a preferable strategy is to deliver membrane protein into the anionic target bilayer, which provides a better lipid environment for activity of membrane proteins (discussed further in the text).

The GUV formation protocol with inverted emulsion method is highlighted in detail in Figure 2. We prefer to use hexadecane in the lipid-oil mixture due to its relatively high (18 °C) freezing temperature, which allows easy removal of solidified oil after GUV pelleting.

Figure 3 demonstrates vesicle fusion using cobalt-calcein-EDTA method. Fusion is seen only when complementary charged vesicles are used in low salt buffers, while higher salt concentrations (>50 mM^14^) or the use of non-fusogenic vesicles demonstrate no fusion.

This fast protocol of reconstitution of membrane proteins into fusogenic SUV is illustrated in Figure 4A. Using this protocol, we demonstrate fast reconstitution of primary proton pump bo_3_-oxidase and F_1_F_o_ ATP synthase into PL, and assessment of their specific activity in these membranes. It is important to mention that the yield of protein reconstitution does not depend on the charge of a lipid used^15^ and is about 50 - 75%^25^,^12^, and that after reconstitution into PL, the protein can be stored for at least three days, even at room temperature without obvious loss of protein activity. This procedure also provides unidirectional orientation of ATP synthase, where more than 95% of it has its hydrophilic moiety F_1_ oriented outwards^15^,^26^.

Figure 5 demonstrates that the proton pumping activity of F_1_F_o_ (Panel A) and bo_3_-oxidase (Panel B) in cationic lipids is reduced and sensitive to low ionic strength, as compared to anionic and neutral lipid environment, but this effect is mitigated in postfusion membranes after fusing PL^+^ with anionic LUV^-^.

Figure 6 demonstrates delivery of F_1_F_o_ ATP synthase into membranes of large vesicles. In this experiment, PL^+^ and 800 nm LUV^-^ were fused in 20 mM KCl for 5 min, and the reaction product was pelleted to remove unfused PL^+^, resuspended and assayed for proton pumping. Control experiments showed no proton pumping when LUV^0^ instead of LUV^-^ were used in fusion reaction (black trace), or empty LUV^-^ alone were assayed (blue trace).

Fast assembly of a functioning electron transport chain in membranes of large vesicles by means of fusogenic PL is shown in Figure 7. We used F_1_F_o_ SUV^+^ and bo_3_ SUV^+^ for fusion with 800 nm LUV^-^, and demonstrated ATP production by this chain in the postfusion vesicles by sequentially adding Coenzyme Q_1_ and DTT to energize membranes, and then adding phosphate to trigger ATP synthesis by F_1_F_o_.

**Figure:**
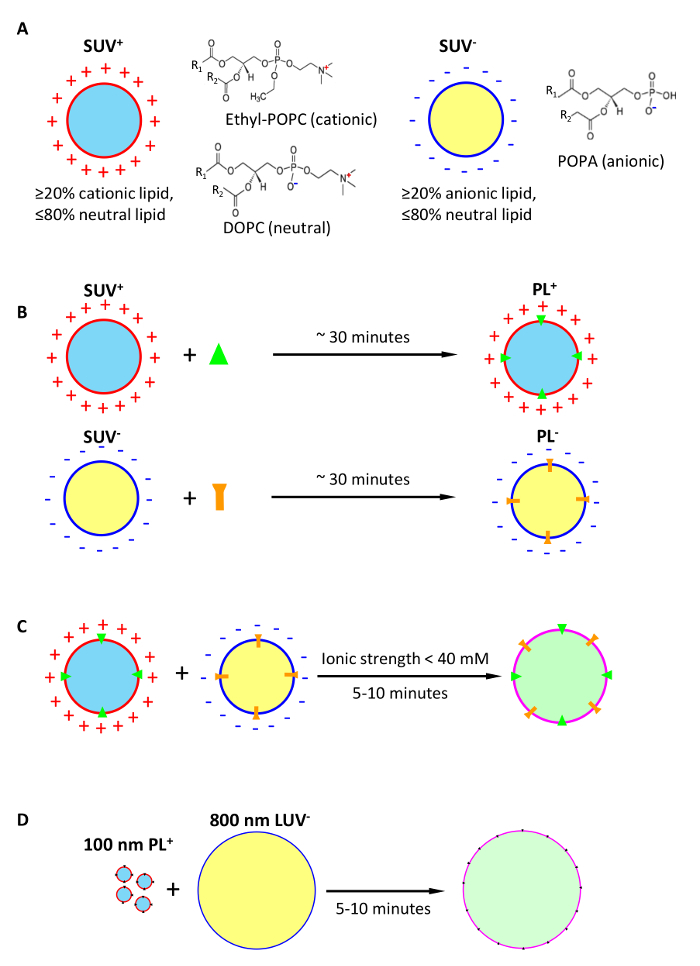

**Figure 1: Conception of ultrafast detergent-free delivery of membrane proteins into target lipid bilayers via fusion of unilamellar vesicles formed of complementary charged lipids.** (**A**) formation of cationic and anionic fusogenic small unilamellar vesicles (SUV^+^, SUV^-^) optionally loaded with intravesicular cargoes. (**B**) Conversion of fusogenic SUV into fusogenic proteoliposomes (PL) by reconstitution of membrane proteins. (**C**) Detergent-free delivery of membrane proteins into postfusion membranes with fusogenic PL. (**D**) Preferable strategy for delivery of membrane proteins into membranes of large vesicles, illustrated by the fusion between 100 nm PL^+^ and 800 nm LUV^-^. Please click here to view a larger version of this figure.

**Figure:**
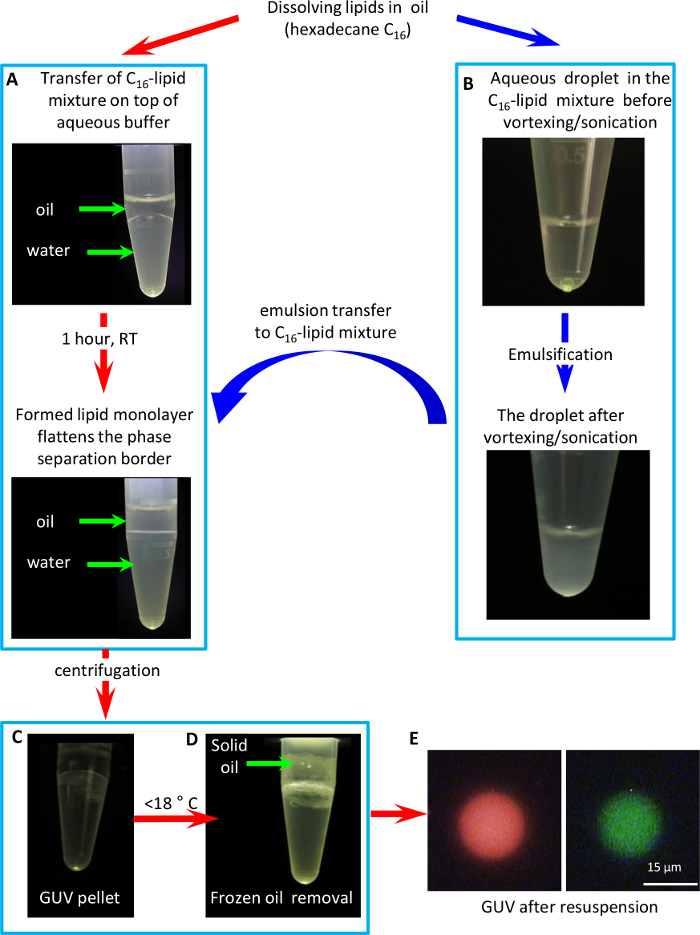

**Figure 2: GUV formation protocol with an inverted emulsion method.** (**A**) Formation of a lipid monolayer on the border between the oil-lipid mixture and water. (**B**) Formation of an inverted (water-in-oil) emulsion. (**C**) GUV formation by passing the emulsion through the oil-water border by means of centrifugation. (**D**) Cooling of the tube below <18 °C to solidify and remove the oil. (**E**) A fluorescent microscopy image of a GUV containing fluorescent lipid (1% weight fraction cholesteryl-Bodipy-FL12) in its membrane (green) and a polar fluorophore (1 mM Sulforhodamine 101) in vesicle`s lumen (red). Please click here to view a larger version of this figure.

**Figure:**
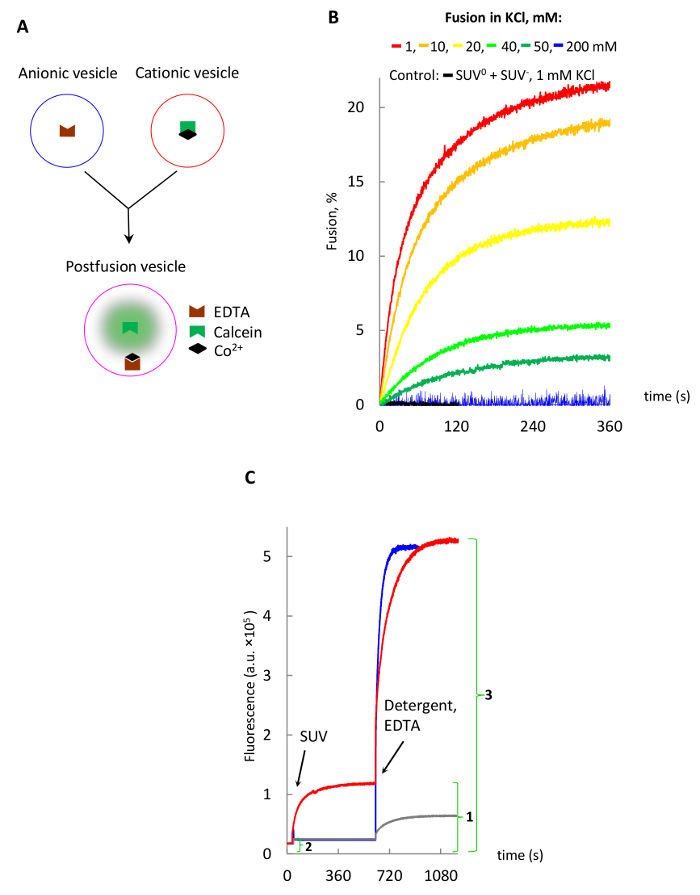

**Figure 3: Lipid vesicles fusion studied with the cobalt-calcein-EDTA method.** (**A**) Schematic of the method, where free calcein is released from a non-fluorescent cobalt-calcein complex by EDTA. (**B**) Fusion of SUV in various KCl concentrations. (**C**) Release of the intravesicular calcein by addition of EDTA and Triton X-100 detergent to postfusion vesicles shown in **B** as described in text. The fusion extent (%) for red trace is calculated by dividing the maximal background-corrected fusion signal (1 - 2) by the background-corrected maximal signal following the release of encapsulated calcein (3 - 2) and multiplying by 100. Please click here to view a larger version of this figure.

**Figure:**
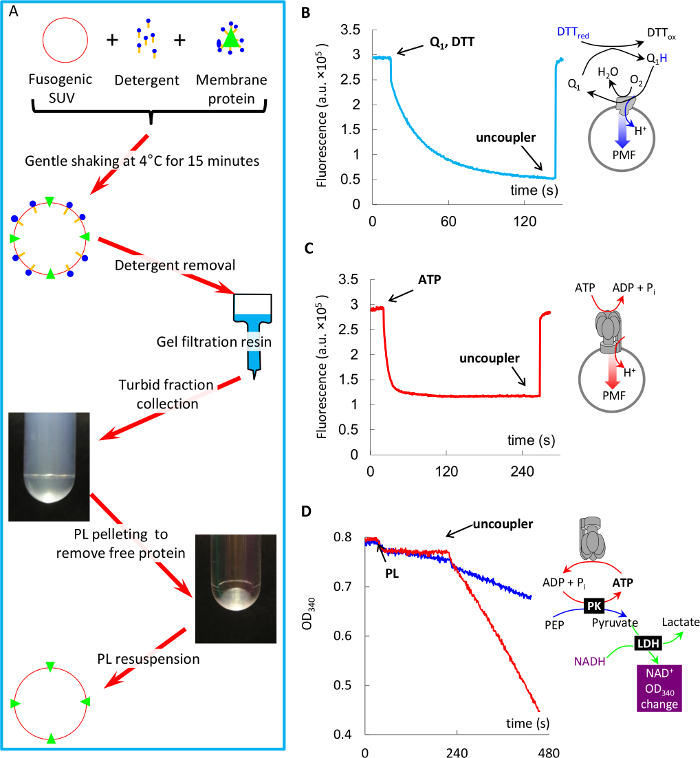

**Figure 4: Ultrafast reconstitution of bo_3_-oxidase and F_1_F_o_ into fusogenic proteoliposomes, and protein activity measurements in such proteoliposomes.** (**A**) Schematic of the reconstitution protocol. (**B**) Coenzyme Q_1_ oxidation driven proton pumping by bo_3_-oxidase in PL measured with ACMA quenching (explained in text). (**C**) ATP hydrolysis-driven proton pumping by F_1_F_o_ in PL measured with ACMA quenching (explained in text). (**D**) ATP hydrolysis by F_1_F_o_ in PL measured with ATP-regenerating system (explained in text), and its stimulation by the uncoupler. Please click here to view a larger version of this figure.

**Figure:**
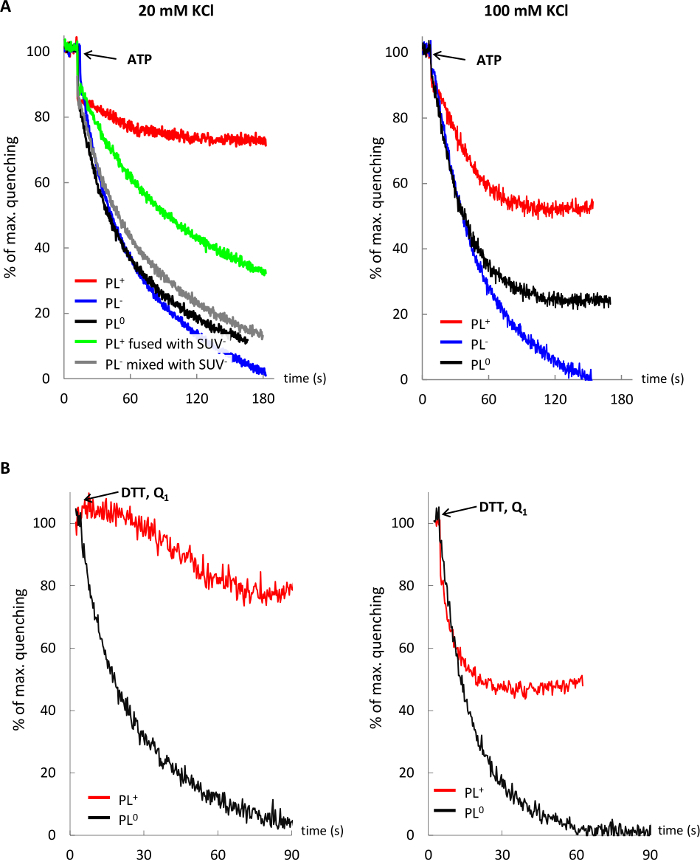

**Figure 5: Influence of the lipid environment and ionic strength on activity of membrane proteins.** Proton pumping by F_1_F_o_ (**A**) and bo_3_-oxidase (**B**) in cationic PL (red trace), anionic PL (blue trace), neutral PL (black trace), and cationic PL fused with anionic LUV (green trace) in 20 and 100 mM KCl. Control trace (gray) shows no change in proton pumping by F_1_F_o_ PL^-^ upon mixing with SUV^-^. Please click here to view a larger version of this figure.

**Figure:**
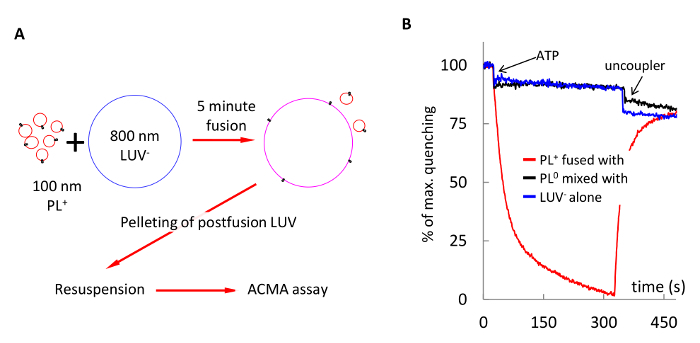

**Figure 6: Delivery of F_1_F_o_ ATP synthase into membranes of 800 nm LUV^-^ via fusion with PL^+^.** (**A**) schematic of the experiment: PL^+^ and 800 nm LUV^-^ were fused in 1 mM MOPS (pH 7.4), 1 mM MgCl_2_, 20 mM KCl for 5 min, pelleted to remove unfused PL, and resuspended in the same buffer. (**B**) Proton pumping by the postfusion LUV (red trace). Control experiments showed no ACMA quenching when PL^0^ were mixed with LUV^-^ (black trace), or empty LUV^-^ alone were assayed (blue trace). Please click here to view a larger version of this figure.

**Figure:**
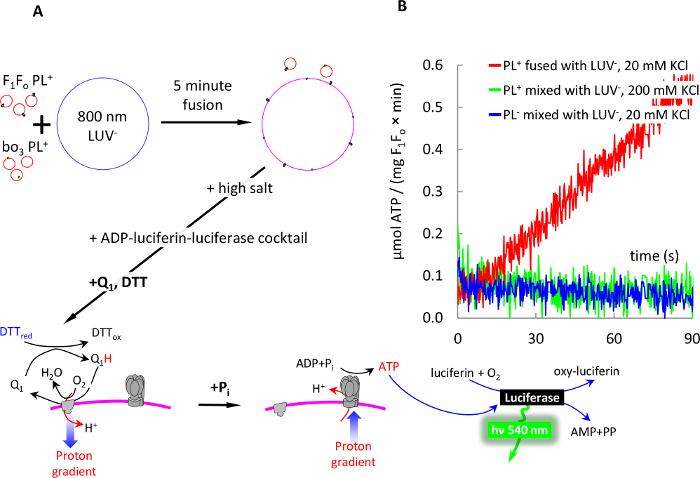

**Figure 7: 5-min detergent-free assembly of an electron transport chain in membranes of 800 nm LUV^-^ via fusion with PL^+^. **(**A**) schematic of the experiment: 100 nm F_1_F_o_ PL^+ ^and 100 nm bo_3_-oxidase PL^+^ were fused with LUV^-^, as described in Figure 6. Fusion was stopped by adding KCl and MOPS to 100 and 50 mM, respectively. The membranes were mixed with ADP-luciferin-luciferase cocktail and energized by addition of DTT and Q_1_, as described in the text. ATP production was initiated by addition of 1 mM phosphate (P_i_) and monitored real time with luciferase-luciferin system as described in the text. (**B**) ATP synthesis by postfusion vesicles (red trace). Control experiments showed no ATP production when PL^+^ were mixed with LUV^-^ in high salt (grey), or PL^0^ were mixed with LUV^-^ (black). ATP synthesis rate was calculated as explained in the text (steps 8.8 - 8.9). Please click here to view a larger version of this figure.

## Discussion

The following few issues need to be considered for success of this experimental approach:

**Choice of lipid charge for proteoliposomes and target bilayers:** Cationic lipids are not found in nature, while anionic lipids are abundant in biological membranes reaching, for example, ~25, 35 and 20% in inner membrane of *E. coli*, plasma membrane of yeast *S. cerevisiae*, and inner mitochondrial membranes of many species, respectively^27^,^28^,^29^. It would be reasonable to expect that the functionality of membrane proteins in PL^+^ may be affected by the strength of a positive charge of the bilayer, which in turn would depend on a relative content of cationic lipid in the bilayer and external ionic strength. Therefore, it is important to address experimentally to what extent functionality of membrane proteins of interest would depend on the charge of the cationic lipid environment. Here, we show that both F_1_F_o_ ATP synthase and bo_3_-oxidase are sensitive to cationic lipid environment, but we managed to modulate and reverse this effect by first placing the proteins in PL^+^ and delivering them into anionic accepting bilayers, and then increasing the ionic strength of the reaction medium after fusion is finished.

**Choice of a particular cationic lipid: **Most commercially available cationic lipids are of non-triacylglyceride nature; therefore a potential candidate lipid must be tested for compatibility with membrane proteins of interest. We found previously^15^ that the ATP synthase used in this study showed its best performance in PL^+ ^formed of ethyl-PC, which has the highest structural similarity to the natural triacylglyceride lipids, while in DOTAP (non-triacylglyceride lipid) PL^+^ this protein was less active.

**Vesicle fusion assays: **True vesicle fusion (when intravesicular liquid contents mix) needs to be differentiated from intermediate hemi-fusion states, when vesicles adhere to each other without mixing their aqueous content, but readily mix contents of their external or both lipid leaflets^30^. In case of suboptimal lipid mixtures or certain conditions, vesicles also demonstrate pronounced liquid content leakage during fusion^31^. Minimal concentrations of charged lipids in fusogenic mixtures enabling true fusion need to be found experimentally for each lipid species, but in general, it is found that membranes with less than 10% charged lipids are rendered non-fusogenic 32.

While forming fusogenic SUV in presence of multi-valent ions, it is important not to mix oppositely charged components, as this may cause immediate clumping and aggregation of lipids by these ions. For example, while preparing SUV for the cobalt-calcein-EDTA method, it is important to avoid mixing a cationic lipid mixture with EDTA to prevent clogging of the polycarbonate filter by clumped components.

It is also important to mention that the cobalt-calcein-EDTA method, while being very sensitive and convenient for real-time fusion monitoring, may still underestimate the extent of fusion due to 1) self-quenching of fluorescence of free calcein inside postfusion vesicles, which is expected to reach 1 mM, while the self-quenching threshold is reported to be around 20 µM^33^, and 2) surface-bound cobalt-calcein, which remains bound to SUV^+^ even after passing through the gel-filtration resin and colors vesicles to a bright orange color, while SUV^0^ have a pale orange color. Also note that release of the bound cobalt-calcein upon addition of detergent Triton X-100 in the presence of EDTA generates much stronger signals for SUV^+^ (Figure 3C, red trace) than SUV^0 ^(grey trace).

**Perspectives**: We expect that the fast approaches described here may greatly facilitate and speed up assembly of complex membranes for the needs of emerging synthetic biology applications. Our membrane protein reconstitution protocol takes only half an hour for reconstitution of fragile large membrane proteins known to be sensitive to lengthy dialysis-based reconstitution techniques, while this fusogenic approach takes only 5 - 10 min to deliver such proteins into large lipid bilayers. Here, we demonstrate the advantages of these approaches by manipulating *E. coli* F_1_F_o_ ATP synthase, which is an example of a fragile protein. It is made of 23 subunits and is known to readily lose its integrity if exposed to suboptimal conditions (for example, heat) during/after solubilization, but being used in these procedures, the protein demonstrates reproducibly high activity in proton pumping.

## Disclosures

The authors have nothing to disclose.
